# “WE DON’T TAKE CARE OF OURSELVES AS WELL AS WE NEED TO.” A QUALITATIVE STUDY OF FAMILY CAREGIVERS’ SELF-MANAGEMENT

**DOI:** 10.1093/geroni/igad104.2590

**Published:** 2023-12-21

**Authors:** Dawon Baik

**Affiliations:** University of Colorado, Aurora, Colorado, United States

## Abstract

Despite the importance of enhancing family caregivers’ (FCGs) physical and mental health to prevent FCGs from becoming care recipients themselves and provide a better quality of care to their care recipients, FCGs are not likely to take care of themselves. In this study, we examined the physical and mental health status and challenges in self-management among older FCGs of persons with heart failure (HF-FCGs). We administered to 15 older HF-FCGs, a demographic survey, the Charlson Comorbidity Index, a health-related quality of life (HRQoL) instrument (SF-36) and conducted semi-structured interviews. Mean age was 65.9±4.4 years, eleven were female, and thirteen were spouses. Chronic diseases were reported among HF-FCGs as; 47% arthritis, 33% obesity, hearing impairment, and visual impairment, and 20% depression and anxiety. HRQoL ranged from 52.3 (±18.8) to 84.3 (±10.8) across the eight subscales. The Energy/Fatigue subscale had the lowest (poorest) score on the SF-36. The challenges in their own health care management were a lack of time and concerns related to symptoms worsening of persons with HF. Most participants were struggling to manage their mental health. They reported stress, anxiety, difficulty staying positive and balancing their mood. Other challenges included managing pain from surgeries (e.g., hip replacement). Many participants felt that they did not take care of themselves as much as they needed, and should receive education to improve their own mental, physical, and social health. Future studies should design and test tailored, caregiver-centered support interventions that enhance health and quality of life in the older caregiver population.

